# Aortic stenosis is a risk factor for all-cause mortality in patients on dialysis: a multicenter prospective cohort analysis

**DOI:** 10.1186/s12882-018-0877-6

**Published:** 2018-04-03

**Authors:** Daijo Inaguma, Yuji Sasakawa, Noriko Suzuki, Eri Ito, Kazuo Takahashi, Hiroki Hayashi, Shigehisa Koide, Midori Hasegawa, Yukio Yuzawa, Tokai Aortic Stenosis in Dialysis Patients Cohort Study Group, Hirofumi Tamai, Shuichi Tsutsui, Takuya Ueda, Yukio Narita, Fumio Sofue, Yasuhiro Hirano, Masahiro Motokawa, Masamiki Miwa, Nobuo Suzuki, Shinichiro Kojima, Hisato Takatsu, Toshiyuki Akahori, Kazutaka Murakami, Yasunobu Shimano, Takashi Miyazaki, Kaori Baba, Yoshiyasu Iida, Haruki Endo, Ryuichi Furuya, Isao Aoyama, Yasuhide Mizutani, Hachiro Seno, Takashi Nagaya, Hirotake Kasuga, Satoshi Sugiyama, Kanako Kojima, Kazuhiro Fujisawa, Tomohiko Naruse, Osamu Ishida, Hideto Oishi, Akira Ono, Hideaki Shimizu, Kiyonari Kato, Isao Ito, Shinji Yasutomi, Chikao Yamazaki, Kaoru Yasuda, Teppei Matsuoka, Yoshinari Tsuruta, Masao Mizuno, Masataka Ono, Masahiro Okada, Akiko Tanoue, Hirotake Kasuga, Takaaki Obayashi, Itsuo Yokoyama, Hiroko Kushimoto, Hiroshi Hasegawa, Masao Kawasumi, Atsushi Nomura, Yasuhiro Sakurauchi, Mitsuru Yamashita, Hiroaki Asada, Keiji Ohara, Sukenari Koyabu, Masashi Tada, Fumihiko Sato, Satoshi Yamaguchi, Hiroshi Ogawa, Yoshihiro Ota, Yoshihiro Matsumoto, Satoki Otsuka, Yasushi Namii, Yasushi Kasai, Nobuo Kato, Makoto Nakayama, Haruo Sato, Shinichiro Inaba, Masaya Shibata, Hiroshi Yamashita, Junichiro Yamamoto, Makoto Yamaguchi

**Affiliations:** 1 Department of Nephrology, Fujita Health University, Toyoake, Aichi Japan; 2 Mizuno Clinic, Toyoake, Aichi Japan

**Keywords:** Aortic stenosis, Dialysis, Mortality, CKD-MBD

## Abstract

**Background:**

Aortic stenosis (AS) is common in patients on dialysis as well as in the general population. AS leads to difficulty with dialysis therapy because of unstable conditions such as intradialytic hypotension due to low cardiac output. However, the precise morbidity rates and risk factors of AS in patients on dialysis are unknown. Moreover, there are no large-scale observational studies regarding the association between AS in patients on dialysis and mortality. Therefore, we will investigate whether morbidity of AS in patients on dialysis is associated with mortality.

**Methods:**

This is a multicenter prospective cohort analysis in the Tokai region of Japan. The 75 participating centers in this study will enroll approximately 2400 patients during 12 months, with or without AS. We started enrollment in July 2017 and will follow patents until June 2023. Transthoracic echocardiography will be performed to evaluate aortic valve. Parameters used for evaluation of aortic valve are mean pressure gradient between left ventricle and ascending aorta, aortic valve area, and maximum aortic jet velocity. We will diagnose AS using the criteria based on the 2014 American Heart Association/ American College of Cardiology Guideline. We will also perform transthoracic echocardiography at 12, 24, 36, 48, and 60 months. Survival prognosis and CV events will be determined at the end of June 2019, 2020, 2021, 2022, and 2023. Development of AS will be also evaluated as new onset or annual change in AS parameters. We will classify patients based on the presence or absence of AS and the stages of AS and will compare outcomes. Study outcomes will include the following: 1) all-cause mortality rates; 2) incidence of cardiovascular (CV) events; 3) CV-related mortality rates; 4) infection-related mortality rates; 5) new onset or development of AS.

**Discussion:**

We will consider the following hypotheses in this study, among others: The prevalence of AS is higher in dialysis patients; new onset and development of AS are associated with factors that are specific for dialysis, such as hyperphosphatemia, hyperparathyroidism, and medication; and outcomes in AS patients are poorer than in patients without AS at baseline.

**Trial registration:**

UMIN000026756, Registered March 29 2017.

## Backgrounds

The number of patients on maintenance dialysis is increasing worldwide. Diabetic nephropathy and hypertensive kidney disease account for most of these patients. Chronic kidney disease (CKD) is an independent risk factor for cardiovascular disease (CVD) [1–5]. Moreover, CVD, which leads to difficulty with dialysis therapy, is strongly associated with higher mortality. Coronary heart disease is a typical condition among CVDs. However, aortic stenosis (AS) is increasingly frequent in patients on dialysis. Previous reports have indicated that onset and progression of AS were related to aging, hypertension, diabetes, or lipid disorders [6–8]. Some epidemiological studies in the general population have shown that the morbidity rate of AS in those over 75 years old is 2–4% [9]. Another general population study involving over 1.2 million individuals in Canada revealed that there were 20,995 patients who needed admission to hospitals or interventions for AS during 13 years of median follow-up [10]. On the other hand, patients on dialysis are more likely to develop AS because of hyperphosphatemia, anemia, and other factors. However, accurate morbidity rates and risk factors of AS in patients on dialysis remain unknown. Moreover, there are no large-scale observational studies of the association between AS in patients on dialysis and mortality or onset of cardiovascular (CV) events. Hence, we will examine whether morbidity of AS in patients on dialysis is associated with mortality, using the database of the Tokai Aortic Stenosis Cohort in Patients on Dialysis. We believe this is the first study to evaluate this association. We will consider the following hypotheses in this study: 1) The prevalence of AS is higher in dialysis patients than in the general population. 2) New onset and development of AS are associated with traditional risk factors including aging and comorbidity of diabetes. 3) New onset and development of AS are associated with factors that are specific for dialysis such as hyperphosphatemia, hypercalcemia, and hyperparathyroidism. 4) Outcomes in AS patients are poorer than in patients without AS at baseline. 5) Outcomes are poorer according to AS stages.

## Methods/design

### Subjects

The study is a multicenter, prospective cohort analysis in the Tokai region of Japan (Tokai Aortic Stenosis Cohort in Patients on Dialysis). We started enrollment in July 2017. The 75 participating centers in this study will enroll approximately 2400 patients during 12 months. Whether or not AS is present, we will include outpatients who are on maintenance dialysis for at least 12 months, aged over 20 years old, who undergo echocardiography every year, and agree with participation in this study.

### Patient characteristics and data at the time of enrollment (baseline)

Baseline is defined as the time at which echocardiography is performed for the first time from July 2017 to June 2018. We will review the following: 1) age, sex, dialysis vintage, and original kidney disease, blood pressure, and resting heart rate; 2) comorbidities including diabetes mellitus and malignancy; 3) medical history including hospitalization because of heart failure within one year, coronary heart disease, aortic disease, stroke, peripheral artery disease, malignancy, and history of parathyroidectomy; 4) medication including renin-angiotensin blockers, calcium channel blockers, β blockers, vitamin D receptor activators, calcimimetics, phosphate binders, and warfarin; 5) laboratory data including hemoglobin, platelet count, and serum albumin, alkaline phosphatase, uric acid, urea nitrogen, creatinine, adjusted calcium, phosphorus, magnesium, intact parathyroid hormone (PTH), ferritin, and C-reactive protein levels.

### Echocardiography measurements

Transthoracic echocardiography will be performed by using commercially available ultrasound systems owned by each facility during enrollment and the follow-up period every year by an experienced technician. All parameters will be assessed with B mode or tissue Doppler imaging. The investigator will be blinded to clinical data. Findings will be confirmed by a cardiologist. The parameters used for evaluation of aortic valve are mean pressure gradient (mPG) between left ventricle and ascending aorta, aortic valve area (AVA), and maximum aortic jet velocity (aortic Vmax). Doppler echocardiographic measurements will include the peak and transaortic mPG using the simplified Bernoulli equation, and the AVA using the standard continuity equation or planimetry method in most participating facilities. Continuous wave Doppler will be used at multiple windows to obtain the maximal jet velocity over the aortic valve as well. In addition to the AS parameters, we will measure various parameters including left ventricular ejection fraction, left ventricular mass index, left atrial diameter, and E/E’. We will also examine aortic valve calcification.

### Definition and classification of AS

We will diagnose AS using the following criteria based on the 2014 American Heart Association/ American College of Cardiology (AHA/ACC) Guideline for the Management of Patients with Valvular Heart Disease: 1) mPG > 20 mmHg, or 2) AVA < 1.0 cm2, or 3) aortic Vmax > 2.0 m/s [11]. We will evaluate stages A to D of AS according to the AHA/ACC Guideline. We will compare patient profiles, laboratory data at baseline, and outcomes including all-cause mortality and CV events among patients 1) with or without AS, and 2) 4 groups by stages.

### Follow-up schedule

Table 1 shows the follow-up schedule. According to baseline characteristics and laboratory data, we will examine variables, which include comorbidities and medication at 12, 24, 36, 48, and 60 weeks. We will also perform transthoracic echocardiography at 12, 24, 36, 48, and 60 months. Survival prognosis and CV events will be determined by sending letters to facilities at the end of June 2019, 2020, 2021, 2022, and 2023. These letters include a questionnaire regarding the categories of CV events, date of onset, and clinical outcomes.

**Table 1:** Research Schedule

Date	Contents
July 1, 2017 – June 30, 2018	Case registration
September 30, 2017	Fixation of baseline data
	Preparation of paper on baseline
July 1, 2018 – June 30, 2023	Transthoracic echocardiography examination every year
	Surveillance of outcomes every year
December 31, 2023	Fixation of final data
January 1, 2024 –	Preparation of papers on outcomes
	Presentation at relevant national and international conferences

### Outcomes

Study outcomes will include the following: 1) all-cause mortality rates, 2) incidence of CV events, 3) CV-related mortality rates, 4) infection-related mortality rates, and 5) new onset or development of AS. CV events are defined as heart failure requiring hospitalization, acute coronary syndrome, coronary artery disease requiring percutaneous coronary intervention, coronary artery bypass grafting, or medication, and stroke or peripheral artery disease requiring hospitalization. CV-related death is defined as death due to heart failure, acute coronary syndrome, aortic disease, or stroke. Infection-related death includes sepsis associated with peripheral artery disease. Development of AS is evaluated as new onset of AS or annual change in mPG, AVA, and aortic Vmax.

### Study organization

There are several stakeholders in this study: a scientific steering committee, 75 clinical centers, a data management center, and an independent committee to evaluate CV events. Independent Outcome Evaluation Committee are planning to monitor and perform source data verification every year.

1. Steering Committee: Daijo Inaguma, M.D. (Fujita Health University), Yukio Yuzawa, M.D. (Fujita Health University), Midori Hasegawa, M.D. (Fujita Health University), Hiroki Hayashi, M.D. (Fujita Health University), Masao Mizuno (Mizuno Clinic)
2. Data Center: Department of Nephrology, Fujita Health University
3. Independent Outcome Evaluation Committee: Shoichi Maruyama, M.D. (Nagoya University), Yasuhiko Ito, M.D. (Aichi Medical University)
4. Biostatistics Adviser: Kunihiro Nishimura, M.D. (National Cerebral and Cardiovascular Center).

### Statistical processing

We will use SPSS statistics version 24 and the Easy R program [12] for statistical processing. Patient characteristics and baseline data will be compared between those with or without AS and those classified by AS stages using an unpaired t-test, analysis of variance for continuous variables, and Fisher’s exact test for nominal variables. We will compare incidence of all-cause mortality, CV events, CV-related mortality, infection-related mortality, and new onset of AS by using the log-rank test for Kaplan-Meier curves. The factors that contribute to all-cause mortality, CV events, CV-related mortality, infection-related mortality, and new onset of AS will be examined using univariate regression analysis. We will conduct multivariate Cox proportional hazards analysis using models with adjustment for some variables including age, gender, and factors that will be extracted as significant variables by univariate analysis. We will also examine factors contributing to annual change rates of mPG, AVA, and aortic Vmax with multiple regression analysis by using models. Continuous variables will be expressed as the mean and standard deviation, or the median and interquartile range, and categorical variables will be presented as a percentage. *P*-values less than 5% will be considered statistically significant.

### Sample size

The necessary sample size will depend on the endpoint being evaluated, although there are numerous endpoints in this study with various magnitudes of incidence rates. In addition, this study will function in part as explanatory research. Therefore, we decided to set the sample size as consecutive cases undergoing echocardiography in a year among participating facilities. We anticipate enrollment of over 2400 cases.

## Discussion

We will show real-world data about the relationship between AS and prognosis in dialysis patients. We consider this the first large-scale, prospective observational study. We planned this study to determine whether the prevalence of AS increases and is associated with poor prognosis among dialysis patients.

### Risk for onset and development of AS in dialysis patients

Traditional risk factors for AS are aging, hypertension, comorbidity of diabetes, and hyperlipidemia [6–8]. In addition, increase in extracellular fluid volume, anemia, and increase in cardiac output due to vascular access lead to increased flow velocity and turbulence across the aortic valve in dialysis patients [13]. These can lead to fibrosis and aortic valve calcification. Stinebaugh et al. reported in that higher resting heart rates in dialysis patients create mechanical shear stress on the aortic valve [14]. Moreover, it is likely that chronic kidney disease-mineral and bone disorder (CKD-MBD) influence onset and development of AS in dialysis patients. Therefore, this study will examine blood pressure and heart rates before and after dialysis sessions, as well as the amount of interdialytic weight gain and hemoglobin level.

### Calcification of aortic valve

Ectopic vascular and heart valve calcification is a typical condition in CKD-MBD [15, 16]. Guerraty et al. demonstrated that decline of glomerular filtration rate was associated with aortic valve calcification after adjustment for classic risk factors [17]. Hyperphosphatemia, hypercalcemia, hyperparathyroidism, and other factors induce vascular calcification, while matrix GLA protein, klotho, magnesium, and other factors inhibit progression of vascular calcification. The mechanism of aortic valve calcification is not as well-understood as that of vascular calcification, although the same mechanism is likely. In addition, there is a possibility that calcium-containing phosphate binders and excess of the active form of vitamin D induce aortic calcification, while calcium-free phosphate binders and calcimimetics inhibit progression of aortic calcification. Accordingly, we will examine laboratory data including serum phosphorus, calcium, and PTH levels. Moreover, we will regularly collect information about medication, including type and dosage of phosphate binders, activated vitamin D, and calcimimetics.

### Diagnosis and evaluation of AS

Echocardiography is used to examine the degree of AS severity by measuring mPG, AVA, and aortic Vmax. In this study, we will perform echocardiography and evaluate AS once a year starting at baseline. Hence, we will examine annual changes in the parameters. Brener et al. have shown that annual change rates in the peak/mean PG and AVA in AS patients were 8.3 mmHg, 6.3 mmHg and 0.14 cm2, respectively [18]. Perkovic et al. demonstrated that the annual change rates of AVA and mPG were larger in dialysis patients than in patients without CKD, i.e., − 0.19 vs. -0.07 cm2, and 4.9 vs. 2.5 mmHg [19].

### All-cause mortality in dialysis patients with AS

Some studies have reported the prognosis of dialysis patients with AS. Miura et al. reported that 167 of 519 severe AS patients, with AVA less than 1.0 cm2, died at a median of 3.5 years [20]. In their study, renal impairment and dialysis were proven to be risk factors for mortality. Raggi et al. showed in their prospective study that the hazard ratio for all-cause mortality was almost doubled in patients with calcification of both the mitral and aortic valves [21]. A feature of our study will be the larger scale as well as the longer follow-up period than in previous reports.

### Treatment strategy of AS patients

According to the selection of treatment including medication, surgical aortic valve replacement, and transcatheter aortic valve replacement (TAVR), comorbidity and general condition will be considered [22–24]. The Society of Thoracic Surgeons predicted risk of mortality score or the EuroSCORE is generally used to evaluate risk for selection of surgical treatment. TAVR is likely to be performed in patients with high risk, including those on dialysis. However, the incidence of complications and mortality associated with treatment have been higher in dialysis patients [25, 26]. Optimal treatment selection by stages of AS in dialysis patients remains unknown. Therefore, we expect that the results of our study will lead to a treatment strategy for dialysis patients with AS.

### Limitations

The present study has the following limitations. First, this is an observational analysis, and there will be some differences in the baseline characteristics and laboratory data among the groups. Second, there will be differences in the type of echocardiographic devices among participating facilities.

## Abbreviations

AHA/ACC: American Heart Association/American College of Cardiology
AS: Aortic stenosis
AVA: Aortic valve area
CKD: Chronic kidney disease
CKD-MBD: Chronic kidney disease-mineral and bone disorder
CV: Cardiovascular
CVD: Cardiovascular disease
mPG: Mean pressure gradient
PTH: Parathyroid hormone
TAVR: Transcatheter aortic valve replacement
V max: Maximum aortic jet velocity
